# WHEN GRANDPA SAYS SOMETHING RACIST: THE ROLE OF AGEISM IN YOUNG ADULT RESPONSES

**DOI:** 10.1093/geroni/igz038.318

**Published:** 2019-11-08

**Authors:** Jennifer Sawyer, Jessica H Helphrey, Leah N Smith, Ben K Mokhtari, Allie M Sandlin, Christopher Reed, Daniel Rodriguez, Michael D Barnett

**Affiliations:** The University of Texas at Tyler, Tyler, Texas, United States

## Abstract

Previous research has found that older adults endorse higher levels of racist attitudes than younger adults. However, little extant research has explored how young adults may respond to an older adult expressing racist views. One factor that may drive young adults’ responses is ageism, particularly stereotypes that older adults cannot handle disagreement or are incapable of changing their views. The purpose of this study was to investigate the relationships between ageism and young adults’ likely responses to an older adult relative making a racist statement. College students (N = 110; 75.8% female) completed an online survey in which they were given a scenario in which an older adult relative makes a racist statement and rated how likely they would be to respond in different ways. Factor analysis of the likely response items found four facets: confront, agree, avoid, and leave. Bivariate correlations found that ageism was associated with higher likelihood of agreeing or avoiding, and lower likelihood of confronting the older adult relative. There was no association between ageism and likelihood of leaving the situation. Young adults higher in ageism may be more likely to agree or avoid because of ageist stereotypes that older adults cannot handle disagreement or are incapable of change, and they may be more likely to agree with the racist statement because they may have higher levels of intolerance toward both older adults and other ethnic groups. Ageism may play a role in how young adults respond to older adults expressing intolerant views.

